# Frontline practitioners’ perspective of the implementation of child protection laws and prevention of violence against children in Maputo, Mozambique

**DOI:** 10.1080/16549716.2025.2609403

**Published:** 2026-01-28

**Authors:** Sérgio Nhassengo, Stela Ocuane Matsinhe, Eunice Jethá, Mathilde Sengoelge, Lucie Laflamme, Asli Kulane

**Affiliations:** a Department of Global Public Health, Karolinska Institutet, Solna, Sweden; b Faculty of Medicine, Eduardo Mondlane University, Maputo, Mozambique; c Center for Health Sciences and Medicine, University for Continuing Education Krems, Krems, Austria; d Institute for Social and Health Sciences, University of South Africa, Pretoria, South Africa

**Keywords:** child protection system, child protection services, children, low and middle-income countries, child interpersonal violence

## Abstract

**Background:**

Frontline practitioners play a crucial role in the implementation and enforcement of child protection laws. Yet, studies on how they experience applying those laws are scarce, not least in the sub-Saharan Africa region where rates of violence against children are persistently high.

**Objective:**

This study provides an insight into the views of frontline practitioners in the implementation and enforcement of child protection laws, with a focus on what they experience as barriers and facilitators.

**Methods:**

Interviews were conducted with 17 frontline practitioners from child protection services in Maputo City, Mozambique’s capital. The interviews addressed barriers, facilitators, gender norms and attitudes and forms of violence. Audio recorded interviews were transcribed, translated, and thematically analysed.

**Results:**

The results resolved around two overarching themes: 1) barriers to the implementation and enforcement of child protection laws, including system-related deficiencies (material, human and organizational); Law related shortcomings (gaps in the content and constraints); and sociocultural norms and attitudes perpetuating violence. 2) facilitators to the implementation and enforcement of child protection laws, including access to resource support from non-governmental organisations and newly established multisectoral mechanism for responding to violence.

**Conclusion:**

Effective implementation and enforcement of child protection laws in Mozambique requires adequate resource allocation and funding to strength child protection services. It may also be necessary to enhance multisectoral coordination and harmonization of child protection laws. Furthermore, adapted evidence-based interventions from the WHO INSPIRE framework may help to improve both the prevention and response to violence against children.

## PAPER CONTEXT

**Main findings:** The implementation and enforcement of child protection laws to protect children is hampered by barriers related to limited resources of all kinds, weak collaboration and not well-integrated child protection services, gaps in the legislation and sociocultural norms. Child protection services in Maputo City rely on support from non-governmental organizations.

**Added knowledge:** This qualitative study provides knowledge on experiences of frontline practitioners in the implementation and enforcement of child protection laws, as well as evidence-based for interventions to strengthen child protection services and improve the performance of frontline practitioners.

**Global health impact for policy and action:** Findings from this study highlight the need for national governments of Mozambique and other sub-Saharan African countries to take accountability for the development of holistic and transparent child protection systems by increasing investments and harmonising current child protection legislation with international standards.

## Background

Countries worldwide have ratified the United Nations Convention on the Rights and Welfare of the Child (UNCRC), taking responsibility to promote children’s rights and protect them against all forms of harm [1]. On the African continent, the African Charter on the Rights and Welfare of the Child (ACRWC) fills a similar function [2] and at the time of writing, it has been ratified by all but three countries in sub-Saharan Africa (SSA): the Democratic Republic of Congo, Somalia, and South Sudan [3]. SSA countries have made significant progress in developing policies and establishing child protection laws to address violence against children. But a range of constraints compromise their effective implementation and enforcement as demonstrated in peer-reviewed cross-country surveys [4,5] and in reports published by international organizations like the UNICEF [6,7] and Save the Children [8], or regional ones such as The African Partnership to End Violence against Children [9] and The Africa Platform for Social Protection [10]. A common source of struggle for the systems in place include a lack of funding and under-resourced services [5,6], rudimentary or absent data and record-keeping systems [7], fragmented services [6] with weak coordination [4,8] and the lack of ‘visibility’ of children in the systems in place, for example no dedicated ministry for child protection [8,9] or child-targeted programmes [10].

During the past 10 years four SSA countries have systematically assessed their readiness to effectively implement and enforce child maltreatment laws using the WHO Readiness Assessment for the Prevention of Child Maltreatment (RAP-CM) [11]. The instrument measures the readiness across 10 dimensions (each a scale of 1–10) with each score obtained corresponding to a certain level of readiness, and an overall readiness score is calculated as the average of all dimensions score [12,13]. South Africa completed this in 2012 [14], Côte d’Ivoire in 2019 [15], Kenya in 2021 [16], and Mozambique in 2023 [17]. While the first three countries reached the pre-planning stage of readiness (level 4 of 10), Mozambique obtained an average score of 5.0 over 10, corresponding to ‘preparation’. These levels are all relatively low and, in all four countries, they were pulled down by a lack of funds and resources, the absence of prevention programmes, and poor political will. South Africa was the only country with high scores on having access to scientific data, albeit the report is from the early 2010s. Compared to the other countries, Mozambique had a higher score on legislation (7.0 out of 10) and knowledge (7.5 out of 10). As those assessments are based on interviews made with high-level stakeholders, the results call for a perspective that would shed light on what actually happens in the daily life of frontline practitioners whose responsibilities is to implement the laws, respect children’s rights and enable them to effectively engage in child protection work. As frontline practitioners play a pivotal role in promoting children’s physical and psychological well-being [18], this knowledge is key to sustainable action plans in child violence prevention. In principle, their interactions with individuals, families, and communities can influence change in norms, attitudes, and harmful practices [19].

There are in fact some SSA countries who proceeded to investigate more closely into the implementation and enforcement of child protection laws and policies, among others Ghana [20], Kenya [21,22], Ethiopia [23], South Africa [24], Zimbabwe [25], and Nigeria [26]. The studies reveal cross-cutting intrinsic issues that impede responsive service delivery and echo those reported above, including a lack of overall funding [20,21,26] and shortages in human or material resources [20,24]. But other compromising challenges include contextual factors that are difficult to change such as community awareness [22,24] and social norms and attitudes [23,25]. It is of note that studies from LMICs from other parts of the world show that they also struggle with these issues, for example a lack of resources and funding in Latin America and Caribbean countries [27], a weak legal system in Indonesia [28,29], sociocultural norms in Oman [30] and poor law enforcement and awareness in the Pacific island countries [31]. In Mozambique, the context of this study, there is a growing body of knowledge on barriers encountered by frontline workers in closely related areas, but not specifically on child issues; they are mainly investigations relative to domestic violence in general [32] or sexual violence against children in particular [33,34]. They point to an unavailable national database, untrained staff including health care providers and inconsistencies between guidelines and protocols [32]. These studies reveal weaknesses in the facilities in place, basic resources that are not available, poor and ineffective coordination among services and agencies, and largely counteracting sociocultural norms [33,34]. As underlined, these studies do not address violence against children in all contexts [32] or forms [33,34]; some also are outdated [33,34] or of small size [32]. To help fill these gaps in knowledge, the overarching aim of this study is to obtain a deeper insight in the perspectives of frontline practitioners regarding the implementation and enforcement of child protection laws, with a focus on what they experience as barriers and facilitators.

## Methods

### Study design

This was a qualitative research study.

### Study context and setting

In Mozambique the child protection laws in place include the Constitution of the Republic, Law on Promotion and Protection of the Rights of the Child (Law No. 7/2008), Family Law (Law No. 22/2019), Penal Code (Law No. 24/2019), Civil Registry Code (Law No. 12/2004), Law against domestic violence (Law No. 29/2009), and Law on Prevention and Elimination of Premature Unions [33,35]. In 2012 a multisectoral mechanism was introduced by the government that defines the coordinated response to violence between the Ministry of Health, Ministry of gender and Social Action, Ministry of the Interior, Ministry of Justice and Ministry of education [36]. One-stop service was established that integrated service centres for victims of domestic and gender-based violence to ensure protection and access to all services (health, social, psychological and judicial service) (Decree No. 75/2020).

This study was carried out in Maputo City, which is both a province and the capital of Mozambique. Maputo City is situated in the extreme south of the country and occupies an area of 346 square kilometres, and has urban, semi-urban, and rural characteristics [37]. It has a population density of 3245 inhabitants per square kilometre, with an estimated population of 1,112,607; among them, 445,598 (40%) are children and adolescents (0–18 years old) [38].

Maputo City has seven districts divided into 73 neighbourhoods [38]. The districts have competencies in areas such as local economic development, environmental protection, education and health, public safety, and natural resource management [39]. For this study, two district municipalities from Maputo City were selected: KaMavota, the most rural, has the main food production centres and according to the Ministry of Health (MISAU) report, registered in 2021 (latest data available) 470 cases of violence, including physical, sexual, and psychological. Nlhamankulu, is an urban setting, one of the most populated municipalities and host of the main commercial centre, with registered 1021 cases of violence according to the same year of reporting [40].

### Participants and recruitment

Government efforts to combat and prevent violence against children at the community level are led and coordinated by healthcare units, social welfare, schools, the police, the institute for sponsorship and legal aid, and community-based organisations [41]. On this basis, we purposively selected a total of 12 services (ten governmental and two non-governmental) mandated in child protection and the application of the law in both municipalities, and in each given service, one to two professionals are most directly responsible for that. Participants included were frontline practitioners tasked directly with ensuring child protection and the implementation of the laws and included social workers, police officers, healthcare professionals, teachers, public defenders, and psychologists. From each service, we initially intended to include two participants to ensure richness and diversification of the data. However, in some agencies, only one professional was allocated. The non-governmental organisations (NGOs) included in this study are dedicated in socio-development and health projects such as education, human rights, health and nutrition, and vocational training; they all have a special focus to support children, adolescents in risk situation and their caregivers. Table 1 present the final sample size (*n* = 17) and the characteristics of the participants.

**Table 1.** Characteristics of study participants (*n* = 17).

Characteristics	Categories	Total number
Sex	Men Women	6 11
Age group (in years)	20–29 30–39 40+	2 6 9
Occupation	Police officer Healthcare worker Public defender Educator Social work Psychologist (NGO) Medical doctor (NGO)	4 2 3 4 2 1 1

### Data collection

The interview guide was developed by the research team, adapted from a previous study from Mozambique mentioned above [32], thereby including three questions on violence against children, six on barriers and facilitators of implementation and enforcement of child protection laws, six on attitudes and practices towards violence against children and the role of gender and forms of violence in child protection; and prompts. The interview guide was piloted on two frontline practitioners, and minor adaptations were made. All interviews were conducted individually in Portuguese, audio-recorded, transcribed verbatim with the permission of the informants and field notes were also used to gather more information. Data collection took place between May and June 2024 and conducted by SN and SOM. The duration of each interview ranged from 40 to 60 minutes each. Data saturation was achieved as no new information or ideas were obtained after completion of the 17th interview and researchers were satisfied with the collected data [42]. Although it is a small number of participants, this is all potential people working in those positions.

The central office of the two district municipalities was contacted for their authorisation to conduct the study. Once the authorisation was granted, we requested permission from identified services to approach frontline practitioners. Upon permission, the service-specific frontline practitioners were approached face-to-face in their workplace, explained the purpose of the study, and asked to sign an informed consent form before proceeding to the interview. All potential participants approached accepted to participate in the study.

### Data analysis

Data were analysed using thematic analysis with an inductive approach [43] following the six stages: familiarising yourself with your data, generating initial codes, searching for themes, reviewing themes, defining and naming themes, and producing the report. After listening to the audio records, the interviews were transcribed by SN and SOM and imported into NVivo software version 14. Initial inductive descriptive codes and respective categories were created by SN and reviewed by the research team to develop a coding framework (Supplementary material 1).

Potential themes were identified by SN by combining different codes to form an overarching theme, and final themes were developed iteratively during research team discussions. Initial analysis and interpretation were discussed by SN and LL. After adjustments and agreement on the interpretation, this was then shared and discussed with the other authors.

Analysis involved a constant moving back and forward between the entire data set, research questions, code extracts, themes, and transcript quotations to ensure the reliability of the findings. The research team had a multidisciplinary background, which allowed them to reflect on the interpretation of the data in various ways. The consolidated criteria for reporting qualitative research checklist (COREQ) [44] was used to improve the methodological coherence and reflexive openness of the reporting of qualitative studies (Supplementary material 2).

### Reflexivity

Various steps were taken to maximise the integrity of the study findings. The two researchers SN (male) and SOM (female) involved in data collection are PhD students and have master’s degree in public health. SN is a sociologist, and SOM is a forensic doctor. No relationship was established between the study participants and the researchers prior to the start of the study or after the study and participants did not have any information about the researchers. Before conducting the interviews, participants were informed that the interviewers are doctoral students and the study is part of SN’s thesis, the findings of the study will be published in an open access journal, and it will be used only for that purpose. All researchers were involved in the coding strategy, the development of the final themes and in the analysis process.

### Ethical considerations

Ethical approval was obtained from the Mozambique Institutional Bioethics Committee of the Faculty of Medicine and Maputo Central Hospital (CIBS FM & HCM/024/2022). Participants were informed about the purpose of the study, participation was voluntary and they could withdraw at any time. Written informed consent was obtained, and methods were in place to minimise the risk of breach of confidentiality and anonymity during the data collection, analysis, and reporting.

## Results

Two overarching themes were identified: (1) barriers to the implementation and enforcement of child protection laws and (2) facilitators to the implementation and enforcement of child protection laws, reflecting our main findings. These main themes also encompass different subthemes detailed in Table 2.

**Table 2.** Summary of identified main themes and subthemes.

Main themes	Subthemes
1. Barriers to the implementation and enforcement of child protection laws	● System-related deficiencies (material, human, and organization)● Law-related shortcomings (gaps in the content and constraints)● Counteracting sociocultural norms and attitudes perpetuating violence
2. Facilitators to the implementation and enforcement of child protection	● Access to resource support from NGOs● Newly established multisectoral mechanism for responding to violence

### Barriers to the implementation and enforcement of child protection laws

This theme incorporates three subthemes through which the frontline practitioners reflected about the constraints when navigating the organisation of the services, i.e. limited resources, gaps in service delivery, and weaknesses in the interaction between services. The barriers identified were common in all services from both district municipalities.

#### System-related deficiencies (material, human and organizational)

##### Shortage of resources

All frontline practitioners expressed experiencing a shortage of resources, in particular material ones (medical supplies, transportation, computers or database for case management, private rooms or child-friendly space, and shelters) as the main barrier in their effort to provide adequate services to child victims of violence. Not having enough personnel was also described as an obstacle by most of the interviewees. In most services represented there was typically only one trained professional attending to the victims during regular working hours, and after 3:30 PM the admitted victims were cared for by staff lacking competence in either child protection or in how to assist child victims of violence. Nevertheless, despite their desire to ensure children receive care, frontline practitioners did not hesitate to use their own resources.

… sometimes we don’t have even a pregnancy test, and the victims have to buy it, the ones that don’t have the money to do so, we try to help. (FP #9_Healthcare professional)

##### Gaps in service delivery

Frontline practitioners also experienced challenges delivering adequate health, social, psychological, and legal assistance to victims due to a response system not well integrated and geographically far apart. In that context and given that child victims of violence may have a variety of needs, they must be referred to different services to receive assistance and care. The fact that services of the like are not close to one another was seen as a constraint to providing a timely response, as well as a cause of distress and financial burden for both victims and caregivers. It was also a limitation for frontline practitioners in their effort to ensure effective follow-ups. Additionally, in their view, the existence of poor facilities and a lack of infrastructure impeded a swift delivery of services and care to those children.

… after we receive a victim, we must refer them to other services, which are sometimes far from one another, and it’s stressful for the victims and caregivers and involves costs for them. (FP_#3_Police officer)

##### Weak interaction within services

Several interviewees expressed concerns that the collaboration between services was poor. More specific comments concerned the police and justice services. Weak communication and poor information sharing between services were also mentioned as a deterrent to the provision of comprehensive services. Additionally, it was put forward that there were conflicting perspectives between services concerning their respective roles.

… it’s not easy to work with the police … they don’t like our presence at the police station. You can go there, looking for information about a case and they don’t give any. They don’t see us as partners; they think that we are making their work difficult when defending victims’ interests. (FP_#14_Public Public defender)

#### Law related shortcomings (gaps in the content and constraints)

##### Legal gaps and constraints

Frontline practitioners unanimously raised the existence of an imbalance between what the legislation on child protection demands and how it is implemented in practice. They alluded to the fact that, while many laws of importance are in place, their application and enforcement remain uncommon, which, in turn, gives way to a constant violation of the rights of children. Some interviewees saw the limitations and inconsistencies in child protection laws as major hindrance to the effective implementation and enforcement of the laws. They generate conflicting interpretations of the laws that both benefit the perpetrators who go free and give way to corruption. In addition, some mentioned that this undetermined timelines for the juridical handling of cases within the court system, alongside common delays in legal processes, which often led to an abandonment of the claim by the victims and their caregivers.

The law itself is not clear, has gaps, and opens the possibility for everyone to do whatever they want. Further, the legislation regarding child protection has inconsistencies, while the law 7/2008 on the promotion of children’s rights sets the age at 18 years old. On the other hand, the civil code defines that the age of majority is 21. But Family Law has an article that makes an exception for a girl at the age of 16 getting married with her parents’ consent. (FP_#4_Public defender)

An additional legal constraint raised by police officers, health professionals, and public defenders was related to difficulties in proving that the violence occurred, not least sexual violence. While physical injuries constitute evidence to prove assault, most victims reported the assault days after perpetration, so that when examined by a forensic specialist significant injury may have become less obvious. They also expressed their concern about the impunity of the perpetrators; most of the perpetrators of physical violence are not punished, and for those who are, either the sentence is short, or it is possible to pay a fee to go free. They also shared the view that the balance between the crime of sexual violence and the sentence when it occurred was not proportionate; they were hoping for a revision of the penal code for more severe punishments.

The legislation should be strictly enforced. The sentences for sexual violence against children should be more severe, from 10 to 16 years without the option to pay a fine, because the traumas last forever in the lives of the victims. We must look out more for the little ones and their well-being. (FP_#4_Public defender)

##### Knowledge deficit of the law and protective services by the community

It was also put forward that there is poor dissemination of child protection laws, mainly in rural areas, which contributes to its weak implementation and enforcement. According to frontline practitioners, the lack of knowledge among children and their relatives about child protective laws often leads to resolving cases of child violence in the community rather than through formal mechanisms.

The level of dissemination of the laws in the community is low. Communities are the ones that violate children’s rights and the first ones that must protect children. (FP_#11_Pedagogical director)

#### Counteracting sociocultural norms and attitudes perpetuating violence

The interviewees described sociocultural norms and attitudes towards violence against children as complex and powerful obstacles to the implementation and enforcement of child protection laws. In their experience, violence against children is often conceived as a family matter, rooted in beliefs, gender inequalities, power relations, fear, stigma, and secrecy. In this context, ensuring child protection and/or protecting them from revictimization are not straightforward tasks. Perceptions and attitudes regarding child upbringing were also seen as a deterrent to a good understanding of what the laws entail. More specifically, child corporal punishment is widely accepted and regarded as a means of discipline and behavioural control, instrumental to ensuring that adults are obeyed and respected.

… in the community, violence against children is not a crime. It’s difficult for us to intervene when families see children as their properties, and usually parents question us if we want to teach them how to raise their kids. (FP_#16_Social worker)

There were also instances when frontline workers expressed opinions in favour of corporal punishment. According to them, it was acceptable to use corporal punishment against children for the reasons exposed above.

Hitting at some point is not bad, it depends on the reason. Sometimes we must use physical punishment to make it clear that what he/she did is wrong. We grew up and educated in that way, and I trust we became more mature and respectful. (FP_#13 _Police officer)

Frontline practitioners also mentioned witnessing a lack of cooperation from the families involved and the community representatives when following up and aiding the victims. They reported instances where victimised children are either told not to talk to the representatives of protection services or hidden from them. And reported it was also common that the families of the victimised children, to protect their reputation, would opt to deal with the situation informally with the perpetrator.

Parents often send the child victim of violence to spend some time in a relative’s house so we can’t have access to them. (FP_#16_Social worker)

### Facilitators to the implementation and enforcement of child protection laws

Although the implementation and enforcement of child protection laws is slowed down by the barriers mentioned above, frontline practitioners identified factors that contribute positively to their daily work. This theme is formed by one subtheme stressing the importance of NGOs’ support in addressing service challenges and another underlining a conducive working environment.

#### Access to resource support from NGOs

##### Engagement of NGOs

The crucial role played by the NGOs in implementing and enforcing child protection laws was raised by all interviewees, who provided wide-ranging examples about that support, for instance, assistance from the NGOs in case management, in ensuring that the child receives health and social care, in training stakeholders in child protection, and in putting into place preventive campaigns and activities in the communities.

Kandlelo (NGO) gives psychological and social support to the victims, they also provide financial assistance and occupational therapy to the child’s family to reduce the child’s vulnerability. (FP_#14_Public defender)

The representatives of NGOs’ interviewed underlined their role not only in supporting services mandated with child protection but also in enhancing cross-service collaboration, mutual support, and exchange of experiences to address challenges encountered in the daily work.

We have a very strong network and have created committees to facilitate the coordination between the sectors and sharing experience, to not depend solely on our organisation. (FP_#8_Project manager)

#### Newly established multisectoral mechanism for responding to violence

##### Joint effort

Notwithstanding the barriers in intersectoral interaction, some frontline practitioners expressed that, to some extent, there is cross-service cooperation in managing cases of violence against children. According to them, this cooperative and coordinated environment was sparked with the introduction of a multisectoral mechanism for response and prevention of domestic violence. Some expressed the involvement of the community leaders as a facilitative factor to enforce and implement child protection laws; community leaders have a strong influence in the communities, and working with them helped in disclosing cases of violence against children, creating family- and community-based awareness and providing safety and care for victims.

The multisectoral coordination ends up demystifying many taboos, and the fact that the police, school, healthcare unit, IPAJ, prosecutor’s office, and social action are entry points for reporting ends up facilitating access to services for the victims and families. (FP_#12_Social worker)

##### Operational tools

Frontline workers revealed that available child protection guidelines and protocols facilitate the process of service delivery to children who are victims of violence or at least serve as guiding principles in their work.

I would say that the fact that we have instruments or guidelines on how to manage cases of violence against children is itself a facilitator because we didn’t have it before. (FP_#17_Police officer)

##### Change of mindset regarding violence against children

Frontline workers recognised that attitudes to violence against children have been changing within the profession. For instance, in some working environments, corporal punishment is not approved of, and some frontline workers have the will to report child victimisation to the authorities or intervene against it.

## Discussion

Our interviews with community-level frontline practitioners shed light on three types of barriers they struggled with (Theme 1) and two types of facilitators in their efforts to apply and enforce existing child protection laws (Theme 2). The barriers pertained to 1) system-related deficiencies, human, material and organisational; 2) law-related shortcomings, in content and awareness; and 3) counteracting sociocultural norms and attitudes. The facilitators included 1) access to resourceful support from NGOs; and 2) newly established multisectoral mechanism for response to violence.

The barriers identified in the specific context of Maputo City align well with those put forward in similar studies, either from Mozambique or other countries from SSA. In Mozambique, they echo what was revealed in interviews with frontline professionals working with cases of domestic violence [32] or cases of sexual violence against children [33]. This is not surprising given how closely related violence against women/mothers is to child violence, how overlapping the resources are, and how violence protection systems are established in the country. In other words, resource constraints issues, ambiguities in the laws and conservative norms and values altogether hinder the work of frontline workers in their effort to prevent and protect from violence those vulnerable groups that are most in need, like women and children. The barriers are also in line with what frontline workers from other SSA countries have identified when questioned about their practice [4,14,20–22,24,26]. In fact, in Mozambique, those barriers formed part of what resulted in the country’s low level of readiness to prevent child maltreatment as assessed using the WHO RAP-CM [17].

What this study adds in that respect is that, from the frontline workers’ perspective, the NGOs active in the country play an important role in minimizing the effects of resource-related barriers. In Mozambique, NGOs have been at the forefront to advocate for and contribute to the implementation of both child protection law and social policy reforms to safeguard children’s welfare and rights [45,46]. Several frontline workers in this study experienced that their services rely on partnering with NGOs to have access to resources, make ends meet, and enhance their institutional capacity. It is of note that these organizations are present in many other SSA countries where they make a significant contribution as well, similar to international donors [4,5,47]. It is essential to encourage the continuity of NGOs in child protection work and other child welfare related areas, in combination with the government taking responsibility for the child protection system to ensure long term sustainability.

From another point of view, it appears that the blurred laws and the counteracting sociocultural norms, attitudes and practice tend to reinforce each other’s negative effects from frontline workers. For instance, norms that legitimize harmful practices against children, such as child marriage, which actually is a crime in Mozambique, and corporal punishment that increases child vulnerability to violence, remain obstacles to the implementation and enforcement of child protection laws and hinder frontline practitioner’s willingness to intervene in such cases [23,25,48]. Likewise, gaps in the laws in place and their poor enforcement make it easier to maintain a violence prone sociocultural context. As indicated earlier though, by sharing some of those values frontline workers themselves become part of this vicious circle and do not fully engage in their duties, for instances for children beaten by e.g. parents, relatives, neighbours or teachers. The reasons for them doing so may be attributed to not only cultural influences but also personal experiences with violence as children [49]. This, in turn, that may contribute to inadequate victim responses, and inefficacy of the implementation of child protection laws [50]. As pointed out by many, increased awareness and advocacy for changes in sociocultural attitudes and practices that support violence are key to reform [4,21,51].

Several participants recognized that the newly promoted multisectoral mechanism for the prevention of and response to violence against children contributed to the creation of a more effective working environment. Strategies of the like find support in previous studies that confirmed the added value of cross-sectoral interventions, co-location of services, and joint protocols [52,53]. These are regarded as crucial to minimising the complexity of the procedures and maximising collaboration between services, thus enabling efficient delivery of services and ensuring the application of child protection laws [52,53]. More and better integrated services serve as a means to facilitate victim access to services [54]. Furthermore, cross-services coordination and information sharing improves case management and service delivery [9,22].

To date the co-location of services for victims of violence is in place in healthcare units in Mozambique, of which four are in Maputo City. Frontline workers were positive and favourable towards these services; the implementation of additional ones may benefit from greater consideration of the barriers found in this study, as well as others from the country [32,54].

In addition, frontline practitioners provided some specific recommendations that can be instrumental in that respect, and useful to help tackle detrimental cultural norms and practices. Among them, allocating more resources and improving the existent infrastructures; increasing community literacy through prevention campaigns in the community, hospitals and schools; involving community leaders and parents in child protection programs; and including children’s rights in the school curriculum.

### Strengths and limitations

One important strength of this study is that the selection of interviewees took into account the potential role of variation in the climate of violence against children experienced at community level and reached out to practitioners from various backgrounds and experiences, which is crucial for this kind of study. It is reasonable to say that the quality of the interviews was good; they were conducted by interviewers knowledgeable of the context and trustable by the interviewees. All interviewees were provided with extensive information about the project and given time to make the decision to participate or not. All interviews were conducted at length, none being interrupted or terminated ahead, and the interviewees spoke openly, resulting in rich interviews.

Although qualitatively satisfying and informative, it is not possible to tell whether the interviewees held back some valuable information, which can be an issue when dealing with such sensitive topics. Also, while the results likely provide a relatively good picture of the situation of frontline workers in different types of districts of Maputo City, it is difficult to tell is the city-wise picture is exhaustive and if it fits that of other parts of the country, some of which can be more conservative and even more poorly resourced. Finally, while the research team members have been reflective about their own views and perspectives throughout the project/study, for understandable reasons, it has not been possible to do something similar with the interviewees. They were questioned about the barriers and facilitators in their work but not to consider how their own values and attitudes could come into play. The knowledge gain from the study is that there are instances when the work of frontline practitioners is shadowed by them sharing a culture that bear risks as regards to child protection.

## Conclusion

Frontline practitioners play a crucial role in child protection; however, their efforts are hampered by a variety of systemic and community-based barriers, including resource and funding limitations, cross-service coordination issues, and sociocultural norms. For the effective implementation and enforcement of child protection laws in Mozambique and other sub-Saharan African countries, governments must invest in child protection services by allocating funds and resources to ensure proper care and protection to child victims of violence. The established multisectoral mechanism and integrated support centres represent an important step in that direction in Maputo City. However, it is imperative to improve coordination, clarify the roles and responsibilities, and increase the availability of the services in the communities. This study also recognises the need to enhance community awareness and advocate for changes in attitudes and gender norms, as well as increase literacy about the rights of children.

## Supplementary Material

Supplementary Materials 1_Codding.docx

Supplementary Materials 2 COREQ_Checklist.pdf

## Data Availability

The datasets analysed in this study can be obtained from the corresponding author upon reasonable request.
