# Supplementary material for: Frontline practitioners’ perspective of the implementation of child protection laws and prevention of violence against children in Maputo, Mozambique
Source: Glob Health Action. 2026 Jan 28;19(1):2609403. doi: 10.1080/16549716.2025.2609403 (PMC12854219; doi:10.1080/16549716.2025.2609403)
Supplement: Supplementary Materials 1_Codding.docx [file ZGHA_A_2609403_SM6188.docx]

Supplementary table 1: Sub-themes, categories and codes describing barriers to the implementation and enforcement of child protection laws to protect children from violence emerging from interviews with frontline workers

| **Sub-themes** | **Categories** | **Codes** |
| --- | --- | --- |
| Shortcomings of the resources and organization of the system | Shortage of resources | - Shortage of material resources - Poor infrastructure and shortage of facilities - Absence of basic rapid tests (pregnancy, prostatic specific antigen, hepatitis tests) - Insufficient human resources |
|  | Gaps in service delivery | - Not integrated response system makes the procedures complex - Limited expansion of service program - Slow response time |
|  | Weak interaction within services | - Poor codified intersectoral cooperation - Poor information sharing between services involved - Communication - Conflicting perspectives |
| Legal gaps, constraints and its knowledge | Legal gaps and constraints | - Gap between the child protective laws and in practice - Inconsistent legal interpretations - Limitations of the laws - Delays in legal proceedings - Poor dissemination and enforcement of the laws - Corruption |
|  | Knowledge deficit | - Community unfamiliar with protection services - Uncertainty of ban corporal punishment - Lack of knowledge of laws |
| Sociocultural norms and attitudes perpetuating violence | Corporal punishment acceptance | - Discipline - Respect - Obedience - Behavioural control |
|  | Family and community dynamics in concealing violence | - Concealment of violence due to economic dependence and social prestige - Adult power over children - Threats - Community and family negotiations - Family and child ignorance |

Supplementary table 2: Sub-themes, categories and codes describing facilitators to the implementation and enforcement of child protection laws to protect children from violence

emerging from interviews with frontline workers

| **Sub-themes** | **Categories** | **Codes** |
| --- | --- | --- |
| Resourceful NGOs support | Engagement of NGOs | - Financial assistance - Social and psychological care - Training all stakeholders - Material aid - Human resources and technical support |
| Conducive working environment | Joint effort | - Cooperation and collaboration between sectors - Involvement of the community leaders - Access to focal points in other services |
|  | Operational tools | - Available child protection guidelines and protocols |
|  | Change of mindset regarding violence against children | - Willing to report cases of child violence to authorities - Disapproval of corporal punishment |
